# γPNA FRET Pair Miniprobes for Quantitative Fluorescent In Situ Hybridization to Telomeric DNA in Cells and Tissue

**DOI:** 10.3390/molecules22122117

**Published:** 2017-12-02

**Authors:** Alexander Orenstein, April S. Berlyoung, Elizabeth E. Rastede, Ha H. Pham, Elise Fouquerel, Connor T. Murphy, Brian J. Leibowitz, Jian Yu, Tumul Srivastava, Bruce A. Armitage, Patricia L. Opresko

**Affiliations:** 1 Department of Environmental and Occupational Health, University of Pittsburgh Graduate School of Public Health, UPMC Hillman Cancer Center, Pittsburgh, PA 15213, USA; aso9@pitt.edu (A.O.); elf51@pitt.edu (E.F.); connorthomasmurphy@gmail.com (C.T.M.); 2 Department of Chemistry, Carnegie Mellon University, Pittsburgh, PA 15213, USA; aberlyou@andrew.cmu.edu (A.S.B.); lisa.rastede@nih.gov (E.E.R.); hhpham@knights.ucf.edu (H.H.P.); 3 Department of Pathology, University of Pittsburgh School of Medicine, UPMC Hillman Cancer Center, Pittsburgh, PA 15213, USA; leibowitzb@upmc.edu (B.J.L.); YuJ2@upmc.edu (J.Y.); 4 Department of Radiation Oncology, University of Pittsburgh School of Medicine, UPMC Hillman Cancer Center, Pittsburgh, PA 15213, USA; 5 PNA Innovations, Inc., 10-N Roessler Rd., Woburn, MA 01801, USA; tumul.srivastava@pnainnovations.com; 6 Center for Nucleic Acids Science and Technology, Carnegie Mellon University, 4400 Fifth Avenue, Pittsburgh, PA 15213, USA

**Keywords:** telomere, γPNA, hybridization probe, fluorescent imaging

## Abstract

Measurement of telomere length by fluorescent in situ hybridization is widely used for biomedical and epidemiological research, but there has been relatively little development of the technology in the 20 years since it was first reported. This report describes the use of dual gammaPNA (γPNA) probes that hybridize at alternating sites along a telomere and give rise to Förster resonance energy transfer (FRET) signals. Bright staining of telomeres is observed in nuclei, chromosome spreads and tissue samples. The use of FRET detection also allows for elimination of wash steps, normally required to remove unhybridized probes that would contribute to background signals. We found that these wash steps can diminish the signal intensity through the removal of bound, as well as unbound probes, so eliminating these steps not only accelerates the process but also enhances the quality of staining. Thus, γPNA FRET pairs allow for brighter and faster staining of telomeres in a wide range of research and clinical formats.

## 1. Introduction

Telomeres are the specialized structures at the ends of linear chromosomes. They consist of tandem TTAGGG repeats, coated with the protein complex shelterin [1]. Telomeres shorten with each round of cell division [2], although this process can be mitigated by the telomere lengthening activity of telomerase holoenzyme [3]. There is a great deal of interest in studying the effects of telomere length and damage as it relates to environmental toxins, oxidative stress, aging, and disease [4,5]. While the upregulation of telomerase can prevent the onset of degenerative diseases [6], it is also often associated with the immortality of cancer cells [7]. Advances in understanding how telomeres influence human health has led to the development of telomere measurement assays to identify and characterize: (1) “healthy” individuals at risk for aging-related diseases; (2) individuals with inherited telomere disorders; (3) pre-cancerous and cancerous lesions; and (4) the efficacy of interventions that reduce stress, or modify aging [8,9].

**Chart 1:**
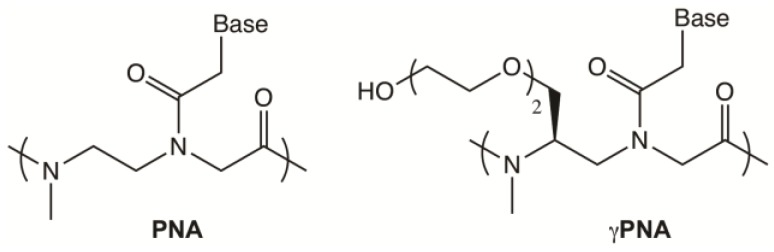
The structure of PNA and γPNA.

There are multiple methods of measuring telomere length and function. Telomere restriction fragment analysis by Southern blot has long been a standard in the field. However, this method normally requires one to three micrograms of intact genomic DNA and only provides an average telomere length for a population of chromosomes and cells [10]. The quantitative PCR based assay offers the advantage of requiring nanogram amounts of genomic DNA that need not be intact, but only reports on the average telomeric DNA content relative to a single copy gene [11]. Currently, quantitative fluorescence in situ hybridization (Q-FISH) using peptide nucleic acid (PNA, Chart 1) probes is the only available method for quantifying the shortest telomeres in intact cells and solid tissue [8]. This method relies on the hybridization of fluorescently labeled PNA probes that are complementary to the tandem nucleotide repeats of telomeres [12]. FISH offers several advantages, including the ability to co-localize telomeres with proteins in intact cells or on chromosome spreads [13,14], quantitate telomere damage or aberrations [15], and measure the lengths of individual telomeres [12,16]. Furthermore, FISH methods of telomere length analysis are scalable to high-throughput platforms for analysis of telomere interphase nuclei [17] or by analysis with flow cytometry [18]. Finally, FISH has been applied to the imaging and quantitation of telomere lengths in tissue preparations [19,20,21].

Although widely used, traditional PNA probes suffer from limitations in their ability to detect critically short telomeres and often require harsh DNA denaturing and wash conditions that can compromise signals and the integrity of co-localizing proteins. In addition, a major limitation of immuno-fluorescent staining of banked tissue is that the background fluorescence or autofluorescence in formalinized and paraffin-embedded tissue is relatively high, which compromises telomere qFISH results [20,22]. Efforts to reduce autofluorescence from lipofuscin, a pigment that accumulates with age, can also dampen signal from immuno-fluorescent staining [23].

An alternative approach to minimizing interference from autofluorescence is to use dyes that have large Stokes shifts, i.e. large differences between excitation and emission wavelengths. Since most endogenous fluorophores have small Stokes shifts, fluorescence from a large Stokes shift dye will be red-shifted away from autofluorescence from the endogenous fluorophore, thereby reducing the background signal [24]. However, this does not address the need for harsh denaturing and washing conditions associated with telomere FISH probes.

Telomere-detecting PNA probes typically consist of three C_3_TA_2_ repeats, complementary to the G-rich strand of the telomere, for a total of 18 monomers [12]. Upon hybridization, this corresponds to one fluorophore every 18 bases of telomeric DNA. Shorter probes offer the advantage of increasing the number of probes per telomere so that more dyes can be delivered to the target for a brighter signal. Previously, we showed that the higher affinity of a backbone-modified version of PNA, known as gammaPNA (γPNA) [25] (Chart 1) can compensate for the shorter probe length, resulting in a 12-mer telomeric γPNA “miniprobe” which yielded brighter fluorescence in the staining of cells with short telomeres, compared to the traditional 18-mer [26]. (The higher affinity of γPNA relative to PNA is believed to arise from its tendency to adopt a right-handed helical structure that is “pre-organized” for binding to complementary DNA [25]).

In this report, we demonstrate how γPNA miniprobes can be used to stain telomeric DNA, without washing to remove unbound probes, while also minimizing interference from autofluorescence. Specifically, we used two γPNAs, designed to hybridize to alternating sites along telomeric DNA. The γPNAs bear donor and acceptor dyes capable of undergoing efficient Förster Resonance Energy Transfer (FRET) when hybridized, due to the short distance between them, whereas unbound miniprobes are too far apart to undergo FRET during the short, excited-state lifetime of the donor. Moreover, FRET results in red-shifted emission from the acceptor probe relative to the donor, resulting in a large effective Stokes shift [27]. Thus, the use of γPNA FRET miniprobe pairs reduces background fluorescence and allows the elimination of harsh wash steps in the FISH protocol, which in turn helps to preserve signal intensity and reduce the time and materials invested in the protocol.

## 2. Results

### 2.1. Design of Telomeric γPNA FRET Pair Miniprobes 

**Figure 1:**
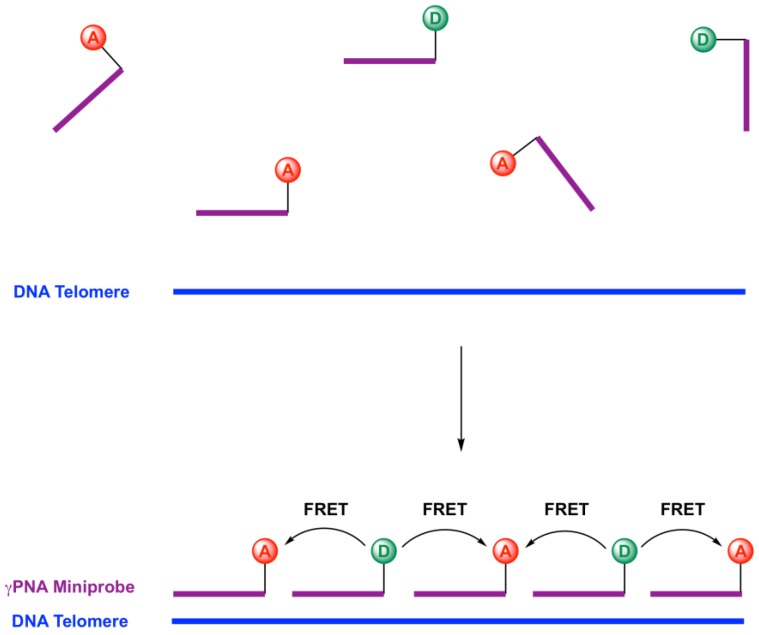
Alternating gammaPNA (γPNA) miniprobes on a DNA telomere for effective FRET signal. D = SCy3 donor, A = X647 acceptor.

FISH methods typically require washing steps prior to imaging, in order to reduce background fluorescence from unhybridized probes. The ability to use relatively short γPNA FRET pair miniprobes provides an opportunity to eliminate the washes. As shown schematically in Figure 1, we designed two 9-mer γPNA miniprobes, that were each complementary to 1.5 telomeric repeats. These miniprobes should hybridize to telomeric DNA in an alternating fashion, positioning each sulfo-Cy3 (SCy3) donor between two Alexa647 (X647) acceptors. This should result in efficient Förster resonance energy transfer (FRET), abetted by the short length of the γPNA miniprobes, which positions the donor and acceptor groups closer together than would be possible for longer probes.

The sequences of the two 9-mer γPNA miniprobes are shown in Table 1. Aligning the two probes end-to-end gives a sequence complementary to three repeats of human telomeric DNA. The γPNAs were synthesized by standard methods (see Materials and Methods). SCy3 was added to the γPNA N-terminus, prior to cleavage from the resin, whereas X647 was coupled in solution after cleavage. Miniprobes were purified by HPLC and characterized by mass spectrometry (Supplementary Materials).

### 2.2. Hybridization of γPNA Miniprobes to DNA Oligonucleotides

**Figure 2:**
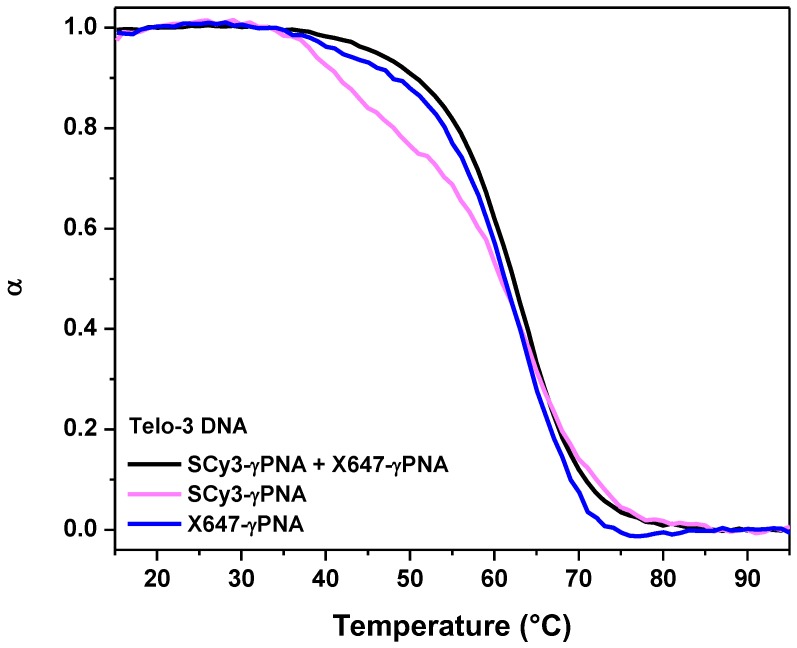
Alpha plots of thermal melting analysis, where α is the fraction in the duplex state. Samples contained 1 µM DNA and 1 µM of each γPNA used.

Each miniprobe forms a stable duplex with its 9-mer DNA complement (T_m_ = 56–57 °C; Figure 2, pink and blue curves). The melting temperature improves by approximately 5 °C when both miniprobes are combined with DNA Telo-3, an 18-mer, on which the miniprobes can hybridize at adjacent positions. We postulate the enhanced stability results from coaxial stacking between the adjacent γPNA miniprobes, as we observed previously for a 12-mer γPNA [26].

**Figure 3:**
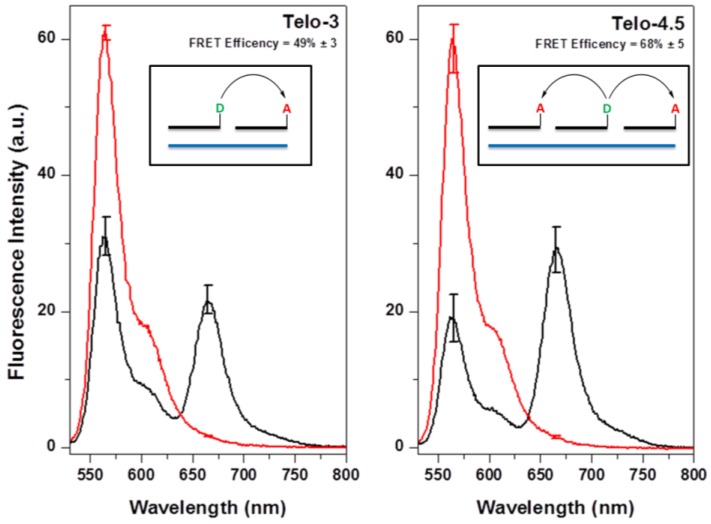
Förster resonance energy transfer (FRET) results for DNA oligonucleotides containing 3 (**left**) and 4.5 (**right**) telomeric repeats. Red curves are samples containing SCy3-γPNA and the unlabeled version of X647-γPNA; black curves correspond to samples with SCy3-γPNA and X647-γPNA with each DNA. Samples contained 200 nM DNA, 200 nM SCy3-γPNA, and either 200 nM X647-γPNA (Telo-3) or 400 nM X647-γPNA (Telo-4.5). Inset is a schematic representation of each DNA (blue) with donor (green) and acceptor (red) γPNAs.

We next used fluorescence spectroscopy to characterize the FRET efficiency. Experiments were performed using DNA oligonucleotides, containing 3 or 4.5 telomeric repeats, in order to confirm that positioning a donor in between two acceptors yields more efficient FRET than having a single acceptor nearby. FRET is evident, based on the decrease in the donor SCy3 peak at 560 nm and the new X647 emission peak at 660 nm (Figure 3, red and black curves). Based on the extent of quenching of SCy3 fluorescence in the presence of X647, we estimate the FRET efficiencies as 54% and 68% when using Telo-3 and Telo-4.5, respectively.

### 2.3. Telomere Staining by FRET γPNA Miniprobes in Human Cells

**Figure 4:**
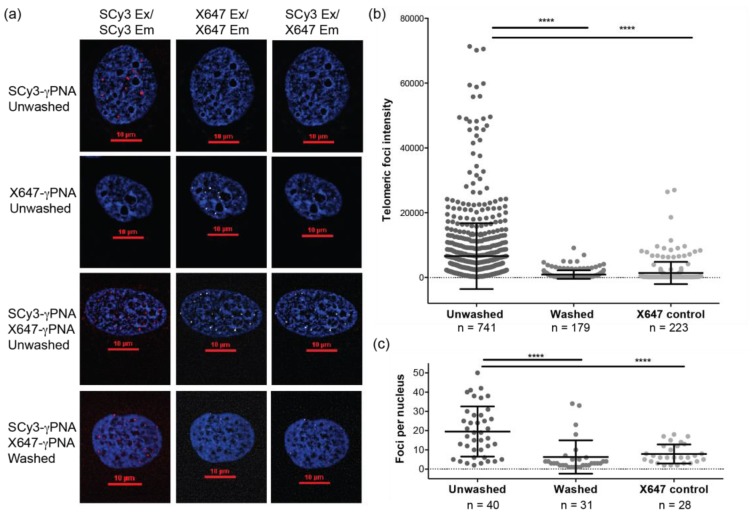
FRET pair miniprobes stain telomeres in human cells. (**a**) Interphase U2OS cell nuclei were stained with the SCy3-γPNA, X647-γPNA, or a mix of both by fluorescence in situ hybridization (FISH), with or without wash steps. Images were captured using filters for SCy3 excitation and emission (red), X647 excitation and emission (white) or SCy3 excitation and X647 emission (white, FRET channel). Nuclei were stained with DAPI and imaged with a 60× lens. Fluorescence signal intensity for each telomeric foci; (**b**) and the number of telomeric foci per nucleus; (**c**) in the FRET channel were measured. Data from unwashed SCy3-γPNA + X647-γPNA (*n* = 40 nuclei), washed SCy3-γPNA + X647-γPNA (*n* = 31 nuclei) and unwashed X647-γPNA alone (*n* = 28 nuclei). Mean and SEM from total foci (**b**) or nuclei (**c**) from three independent experiments are shown. **** *p* < 0.0001, unpaired *t*-test.

To test whether each of the short 9-mer FRET probe could hybridize to telomeres in fixed human cells, we stained nuclei from interphase human U2OS osteosarcoma cells, with each PNA probe separately, in the absence of wash steps. SCy3-γPNA staining revealed distinct nuclear foci upon imaging in the Cy3 excitation/emission settings, and no detectable fluorescence in the Alexa647 (X647) excitation/emission or Cy3 excitation/X647 emission (FRET channel) settings (Figure 4a, top row). Similarly, X647-γPNA staining revealed fluorescent nuclear foci under the X647 excitation/emission settings, but not with the Cy3 channel (Figure 4a, second row). These data indicate that both FRET miniprobes bind to the telomeres, and that there is minimal cross talk between the Cy3, X647 and FRET channels. We next stained fixed U2OS interphase nuclei with a 1:1 mixture of SCy3-γPNA and X647-γPNA. Distinct telomeric foci appeared upon imaging in the FRET channel, indicating efficient energy transfer from the excited SCy3 to the X647 dye on the adjacent probe (Figure 4a, row 3). Importantly, the signal foci intensity and the number of telomeric foci detected per nucleus were significantly higher for samples stained with both SCy3 and X647 γPNA probes, compared to samples stained only with the X647-γPNA probe, upon imaging in the FRET channel (Figure 4b,c). Telomeric foci were also observed in the SCy3 and X647 excitation/emission settings individually (Figure 4a, row 3), suggesting individual probes also perform well in this cell line.

We predicted that the ability to eliminate wash steps would improve staining because inadvertent removal of hybridized probes would not occur. To test this, we examined FRET pair γPNA staining under similar FISH conditions, except we added back the traditional wash steps to remove unhybridized probes. When the nuclei are excited and monitored in the FRET channel, clear telomeric foci are observed, but appear faint compared to the unwashed condition (Figure 4a, compare row 3 to row 4). Furthermore, quantitation reveals that adding wash steps significantly reduces the telomeric foci intensity and the number of telomeric foci detected per nucleus (Figure 4b,c). The presence of more detectable telomeres per nucleus in the unwashed conditions suggests that the ability to eliminate wash steps improves detection of critically short telomeres that may be missed under wash conditions.

**Figure 5:**
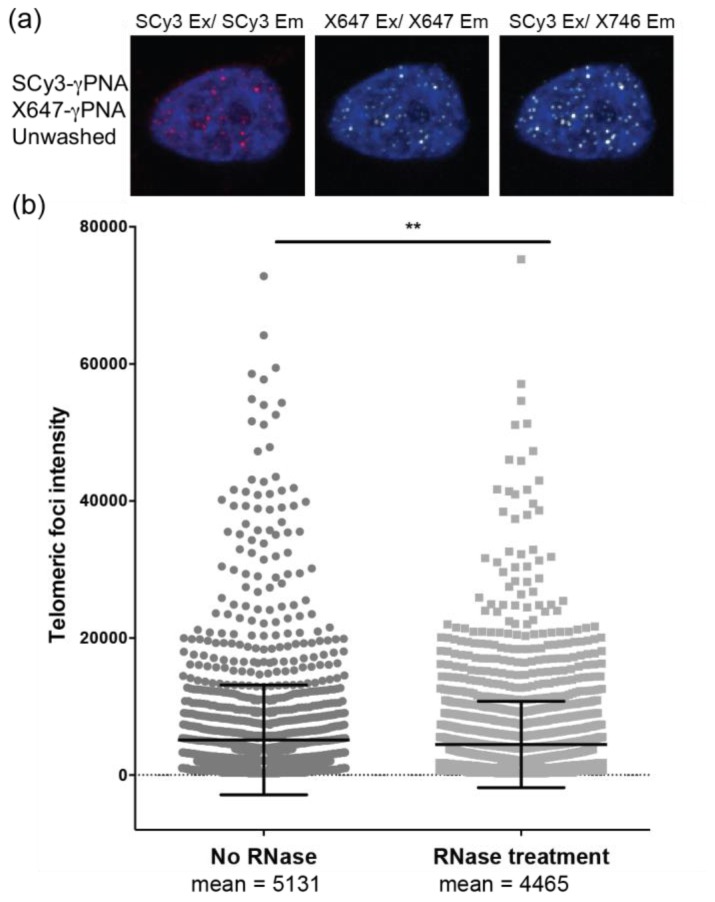
FRET pair miniprobes stain telomeres in human cells with critically short telomeres. (**a**) Interphase HeLa VST cell nuclei were stained with a mix of SCy3-γPNA and X647-γPNA probes (1:1 ratio) by FISH in the absence of washings, and without (shown) or with RNase treatment. Images were captured using filters for SCy3 excitation and emission (red), X647 excitation and emission (white) or SCy3 excitation and X647 emission (white, FRET channel); (**b**) Fluorescence signal intensity was measured for each telomeric foci in the FRET channel. Data from 60 nuclei, each for no RNase (*n* = 1822 foci) and RNase treatment (*n* = 2271), are from two independent experiments. Mean and SEM from total foci are shown. ** *p* = 0.0029, unpaired *t*-test.

To further examine the ability of FRET 9-mer miniprobes to detect short telomeres, we examined staining of interphase nuclei from telomerase positive HeLa VST cells, which possess very short telomeres (average length ~3.7 kb), despite the presence of telomerase [28,29]. We experienced difficulty staining telomeres in these cells with a 12-mer γPNA, due to the very short telomere lengths, and required additional immunofluorescence staining of a telomere binding protein (RAP1), to amplify the telomeric foci signals for detection [29]. In contrast, U2OS cells (Figure 4) are telomerase negative, and use a recombination based mechanism for telomere lengthening, which results in highly variable telomeres lengths [30]. Staining HeLa VST cells with a mixture of the SCy3-γPNA and X647-γPNA (1:1 ratio) in the absence of washes, reveals clear distinct telomeric foci in each channel, but the brightest foci in the FRET channel (Figure 5a). RNase is often included, to reduce background, however, RNase treatment did not further enhance staining (Figure 5b). In summary, FRET miniprobes efficiently stain telomeres in nuclei from telomerase positive and negative cells, and the ability to eliminate wash steps significantly improves signal intensity and detection of short telomeres.

### 2.4. FRET Miniprobes Exhibit Reduced Background Staining on Chromosome Spreads

**Figure 6:**
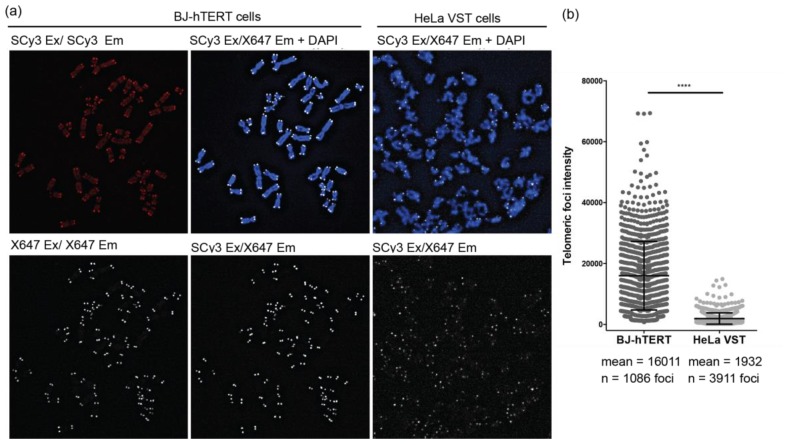
Measurement of telomere length by qFISH on metaphase chromosomes using FRET pair miniprobes. (**a**) Metaphase chromosome spreads from BJ-hTERT or HeLa VST cells were stained with a mix of the SCy3-γPNA and X647-γPNA probes (10:1 ratio) by qFISH in the absence of washing. Images were captured using filters for SCy3 excitation and emission (red), X647 excitation and emission (white) or SCy3 excitation and X647 emission (white, FRET channel). Look up tables (LUT) equal 1000 (BJ-hTERT) or 500 (HeLa VST); (**b**) Fluorescence signal intensity was measured for each telomeric foci in the FRET channel. Data from 5 metaphases for each cell line from 2–3 independent stainings are shown. Mean and SEM from total foci are shown. **** *p* < 0.0001, unpaired *t*-test.

A common application of telomere qFISH is to stain metaphase chromosome spreads and measure the signal intensity at each chromatid end as an indicator of telomere length. Longer telomeres bind more probes and yield a brighter fluorescence signal. We tested FRET pair γPNA staining on metaphase chromosomes, prepared from BJ-hTERT normal cells and HeLa VST cancer cells. BJ-hTERT cells are primary skin fibroblasts, expressing exogenous telomerase that possess normal telomere lengths (~17 kb), much longer than HeLa VST [29]. We found that including traditional wash steps led to the loss of the hybridized miniprobe 9-mer γPNAs. Therefore, all experiments lacked wash steps. In addition, a ratio of 10:1 SCy3-γPNA to X647-γPNA was optimal for staining telomeres on metaphase chromosomes (data not shown). We observed faint telomere foci in the Cy3 and X647 excitation/emission settings, and considerable background staining of chromosomes in the Cy3 channel (Figure 6a, left panels). However, imaging in the FRET channel revealed no background staining, and distinct telomere foci of brighter intensity than foci in the X647 channel (Figure 6a, middle panel). Nonspecific binding typically increases the background signal (SCy3 channel), but the FRET pair probes are unlikely to assemble nonspecifically in a way that would allow energy transfer to occur. We obtained a similar lack of background staining using FRET probes in HeLa VST chromosomes (Figure 6a, right panels). Telomeric foci from HeLa VST chromosomes were significantly less intense compared to foci from the BJ-hTERT chromosomes (Figure 6b), as expected, since telomeres are shorter in HeLa VST cells. This confirms the quantitative capacity of FISH with telomeric FRET miniprobes.

### 2.5. Elimination of Wash Steps Improves qFISH Detection of Telomeres in Tissue Sections

**Figure 7:**
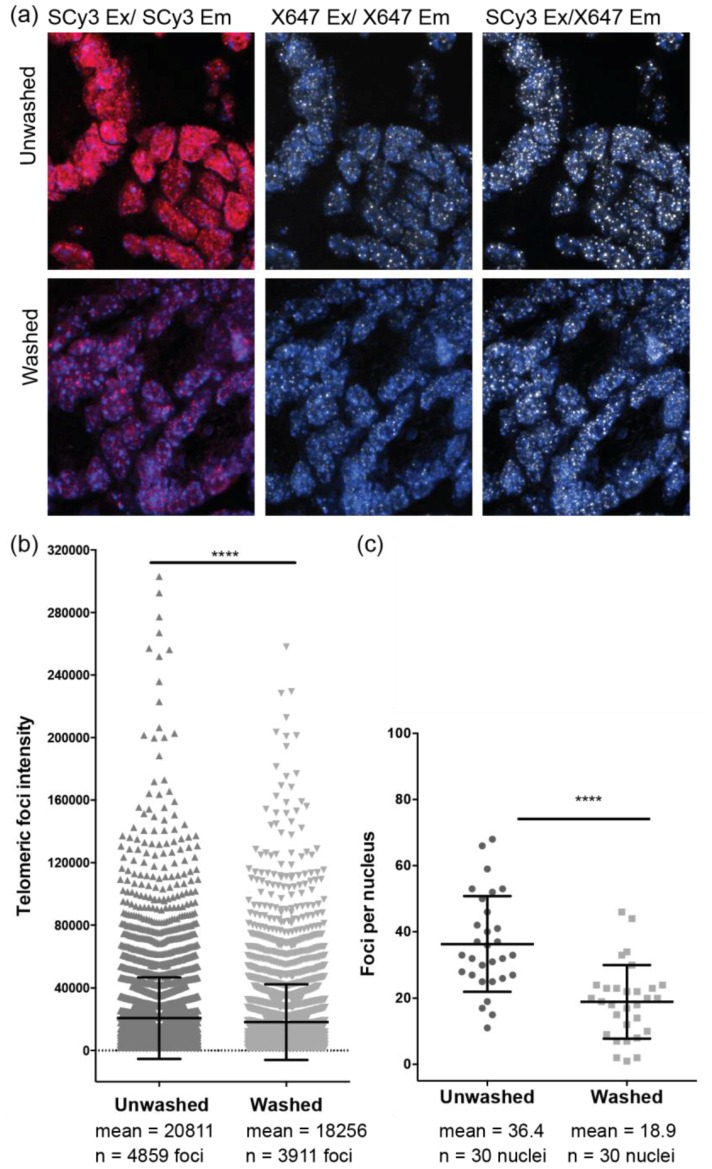
Imaging of mouse intestinal tissue using FRET pair miniprobes. (**a**) Formalin fixed and paraffin embedded section of mouse colon were stained with a mixture of the SCy3-γPNA and X647-γPNA probes (1:1 ratio) by FISH with and without wash steps following hybridization. Images were captured using filters for SCy3 excitation and emission (red), X647 excitation and emission (white) or SCy3 excitation and X647 emission (white, FRET channel). Fluorescence signal intensities for (**b**) each telomeric foci and (**c**) the number of telomeric foci per nucleus were measured in the FRET channel for unwashed and washed samples. Mean and SEM from total foci are shown. **** *p* < 0.0001, unpaired *t*-test.

Next we examined the ability of the γPNA FRET miniprobes to stain telomeres in formalin fixed and paraffin embedded (FFPE) 5 micron sections of mouse colon. Sections were stained with a mixture of SCy3-γPNA and X647-γPNA (1:1 ratio), followed by either no washes, or traditional wash steps, to remove unhybridized probes. Imaging in the Cy3 excitation/emission channel revealed a high degree of background staining that was essentially eliminated upon imaging in the FRET channel. Background staining in the X647 excitation/emission channel was less obvious, but foci were faint compared to imaging in the FRET channel (Figure 7a, top row). Wash steps to remove unbound probes reduced background staining in the non-FRET channels, but also significantly reduced telomeric foci intensity and the average number of telomeric foci detected per nucleus in the FRET channel (Figure 7b,c). These data further demonstrate the benefit of removing wash steps for increasing telomere detection in fixed prepared tissue sections.

## 3. Discussion

The results presented above demonstrate the value of using high affinity γPNA miniprobes for telomere analysis. The ability to use shorter probe lengths enables efficient FRET between 9-mer γPNAs, designed to hybridize in alternating fashion along the length of telomeric DNA. This allowed effective and quantitative telomere staining by the FRET miniprobes on fixed nuclei, metaphase chromosomes, and fixed tissue sections from a variety of sources, including telomerase positive and negative cell lines, and cells with very short telomeres. While the miniprobes could detect telomeres in all three fluorescence channels, we observed reduced background staining under the FRET imaging channel, due to the improved effective Stokes shift [31]. Furthermore, we report that the ability to obtain highly specific staining with protocols that omit wash steps resulted in improved signal intensity and detection of more telomeric foci per nucleus, thus, enhancing the sensitivity of the telomere qFISH assay. While the X647 miniprobe demonstrated a remarkably high performance in telomere staining, the foci intensity was lower compared to imaging mixed probes in the FRET channel.

Telomere dysfunction is a hallmark of early stage carcinogenesis [32]. The finding that pre- and post-malignant tissues show shorter telomeres, compared to normal tissue, in many cancer types [33,34,35,36], has prompted an interest in telomere length measurement in clinical biopsies, as potential diagnostic and prognostic markers in cancer. The FRET pair γPNA miniprobes performed extremely well in quantitative telomere staining in tissue, and could be useful in a clinical setting. Beyond telomere length analysis, a similar approach in probe design may also be useful for quantifying expansion of repeats in trinucleotide repeat disorders, including Huntington’s disease and amyotrophic lateral sclerosis.

Finally, although we have not done a side-by-side comparison, the FRET miniprobe approach to telomere staining described here should be possible with other, high-affinity oligonucleotides, such as locked nucleic acid (LNA) or conventional PNA labeled with suitable energy transfer dyes. While our recent report [26] demonstrated better performance of a 12-mer γPNA miniprobe, compared to an 18-mer conventional PNA probe after washing, it is possible that two PNA 9-mers will have sufficient affinity to stain telomeres in the manner described herein for γPNA and give acceptable signals in a wash-free protocol.

## 4. Materials and Methods 

### 4.1. Materials

DNA oligonucleotides were purchased from Integrated DNA Technologies (idtdna.com) with standard desalting purification. γPNA miniprobe synthesis is described in Supplementary Materials.

### 4.2. Thermal Melting Analysis

The thermal melting analysis was performed using a Varian Cary 3 UV-vis spectrophotometer with a temperature controlled multicell holder. Samples were buffered in 10 mM Tris, 0.1 mM EDTA, 100 mM KCl, pH 7.00 and strand concentrations were 1 µM for each DNA and γPNA. Samples were heated to 95 °C and annealed by slowly cooling to 15 °C (rate = 1 °C/min). The samples were then slowly heated to 95 °C (rate = 1 °C/min). Absorbance was measured at 260 nm. The data are reported as α, or the fraction of DNA/PNA in the duplexed state [37]. Alpha values were calculated by first identifying the upper and lower baselines, corresponding to the fully single-stranded and fully duplexed states, respectively. Upper baselines in Figure 2 utilized absorbance values from 15 °C to 30 °C and lower baselines utilized 85 °C to 90 °C. Next, alpha values were calculated for each temperature, using a ratio of the change in absorbance between the upper baseline and the experimental point and the change in absorbance between the upper and lower baselines for each temperature on the melting curve. Melting temperatures were determined by calculating the temperature where there is 50% duplex and 50% single stranded nucleic acid (i.e., temperature where α = 0.5).

### 4.3. Circular Dichroism (CD) Spectroscopy

CD data were collected on a Jasco 715 spectrophotometer (Jasco Inc., Easton, MD, USA) at 25 °C, using samples previously annealed in UV melting experiments. Each spectrum was obtained by averaging six scans (200–330 nm) collected at 100 nm/min.

### 4.4. Fluorescence Spectroscopy 

Fluorescence data were obtained using a Cary Eclipse Fluorescence spectrophotometer (Agilent Technologies, Santa Clara, CA, USA). Samples were buffered in 10 mM Tris, 0.1 mM EDTA, 100 mM KCl, pH 7.00 and strand concentrations for samples using Telo-3 DNA were 200 nM for Telo-3 DNA, SCy3-γPNA, and X647-γPNA. Samples using Telo-4.5 DNA contained 200 nM Telo-4.5 DNA, 200 nM SCy3-γPNA, and 400 nM X647-γPNA. (Telo-4.5 DNA has two bindings sites for X647-γPNA). Scans were collected at room temperature after samples were annealed (heated at 95 °C for five minutes and then slowly cooled to room temperature). SCy3 was excited at 520 nm and emission was recorded from 530 nm to 800 nm, in order to monitor both SCy3 and FRET emission. Control scans of X647 were excited at 600 nm and monitored from 610 nm to 800 nm.

### 4.5. Cell Culture 

Human telomerase expressing BJ (BJ-hTERT) skin fibroblasts and U2OS osteosarcoma cells were purchased from ATCC (American Type Culture Collection, Manassas, VA, USA). The HeLa VST was provided by Dr. Roderick O’Sullivan (University of Pittsburgh). Cells were cultured in Dulbecco’s modified Eagle medium (DMEM) supplemented with 10% fetal bovine serum (FBS), 50 units/mL penicillin, and 50 units/mL streptomycin (Gibco) at 37 °C in humidified chambers with 5% CO_2_ and 20% O_2_. Except BJ-hTERT cells were cultured with 10% FBS from Hyclone and at 5% O_2_, which improves proliferation of this cell line.

### 4.6. Fluorescent In Situ Hybridization of Interphase Cells

The staining of interphase cell nuclei for telomere foci was based on previous protocols [29] with slight modifications. Briefly, 50,000–150,000 cells were seeded in 35 mm glass bottom dishes (Mattek, Ashland, MA, USA) and allowed to attach overnight. Cells were subsequently fixed in 3.8% paraformaldehyde, washed three times in 1xPBS, then successively dehydrated with 70%, 90%, then 100% ethanol. γPNA miniprobes (final concentration 1 µg/mL each) were added to a hybridization solution (70% formamide, 5% 20× MgCl_2_ buffer, 0.5% blocking reagent, 10 mM Tris HCl pH = 7.5). 20× MgCl_2_ buffer is 20 mM MgCl_2_, 82 mM NaH_2_PO_4_, 9 mM citric acid, pH = 7.4. 10% blocking reagent (Roche 11096176001) is dissolved in maleic acid buffer 100 mM maleic acid, 150 mM NaCl, pH = 7.5. The solution was incubated for 5 min at 95 °C, iced for 5 min, then applied to samples and allowed to hybridize in a dark humid chamber at 70 °C for 10 min, followed by room temperature for 2–3 h. After hybridization the samples were rinsed with PBS once, then counterstained with DAPI, rinsed again in PBS, then allowed to dry. Samples were mounted with ProLong Diamond Antifade (Invitrogen/ThermoFisher Scientific, Waltham, MA, USA) and allowed to cure 24 h before imaging.

### 4.7. Fluorescent In Situ Hybridization of Metaphase Chromosomes

Metaphase chromosome spreads were prepared and stained, as described previously, with some modifications [38]. Briefly, 2 × 10^6^ cells were seeded into flasks and allowed to grow overnight. Cells were then incubated in media containing 0.05 μg/mL colcemid for three hours, then incubated in 75 mM KCl for 12 min (BJ-hTERT) or 7 min (HeLa and U2OS), and fixed in 3:1 methanol: glacial acetic acid solution. Samples were then dropped onto slides, soaked in PBS for 5 min, fixed again in 3.8% paraformaldehyde for 10 min, and washed three times in PBS. Samples were treated with 250 μg/mL RNaseA for 15 min at 37 °C, 1 mg/mL pepsin for 15 min, 3.8% paraformaldehyde fixative for 2 min, and then three rinses with PBS. This was followed by successive dehydration in 70%, 90%, and 100% ethanol. SCy3 and X647 FRET miniprobes were mixed in ddH_2_O in a 10:1 ratio. The mixture was added to the hybridization solution as described above for a final concentration of 1 µg/mL SCy3-γPNA and 0.1 µg/mL X647-γPNA. The solution was heated for 5 min at 95 °C, iced for 5 min, and then applied to samples. Hybridization was at 70 °C for 10 min in a dark humid chamber, followed by overnight at 4 °C. Samples were rinsed three times in PBS with 0.5 µg/mL DAPI in the last wash. Samples were rinsed twice with ddH_2_O, and then successively dehydrated with 70%, 90% and 100% ethanol. Slides were air dried, mounted with ProLong Diamond Antifade (Invitrogen), and allowed to cure 24 h before imaging.

### 4.8. Tissue Fluorescent In Situ Hybridization

The procedures for all animal experiments were approved by the Institutional Animal Care and Use Committee of the University of Pittsburgh. Mice were housed in micro-isolator cages in a room illuminated from 700 h to 1900 h (12:12-h light-dark cycle), with access to water and chow ad libitum. Mouse colons were prepared, as previously described [39]. Briefly, wild-type C57BL/6J mice were sacrificed and colons were removed, opened longitudinally and tacked to a foam board for fixation in 10% (*v*/*v*) formalin overnight. Following fixation, colons were rolled up into “swiss rolls” and embedded in paraffin for sectioning.

Paraffinized samples were heated at 56 °C for 30 min, then deparaffinized by successive rehydration in 100% xylene (10 min), 100% ethanol (10 min), 90% ethanol (5 min), then 70% ethanol (5 min). Samples were added to boiling citrate buffer (8.16 mM sodium citrate, 1.9 mM citric acid, 1 mM EDTA) and then heated for 10 min in a microwave at 10% power. Samples were removed from the citrate buffer and allowed to cool at RT for 15 min. Samples were then treated with 250 μg/mL RNaseA for 15 min at 37 °C and 1 mg/mL pepsin for 15 min, followed by a 30 min PBS wash on a rocking platform. Samples were successively dehydrated in 70%, 90% and 100% ethanol. FRET probes were added to the hybridization solution as described above at a final concentration of 1.5 µg/mL each. The probe solution was heated for 5 min at 95 °C, iced for 5 min, and then applied to the samples. Hybridization was done for 10 min at 78 °C in a dark humid chamber, followed by overnight incubation at room temperature. Samples were rinsed twice with ddH_2_O water, and then three times with PBS. The last wash contained 0.5 µg/mL DAPI. Samples were successively dehydrated in 70%, 90%, and 100% ethanol and allowed to air dry in the dark. Samples were mounted with Prolong Diamond Antifade and allowed to cure for 24 h.

### 4.9. Imaging 

Cells were imaged using a Nikon Ti90 epi-fluorescence microscope (Nikon Inc., Melville, NY, USA) equipped with a PlanApo 60×/1.40 oil immersion objective. Samples were imaged under three filter combinations: (1) Cy3 excitation and visualization of Cy3 emission; (2) Cy5 excitation and visualization of Cy5 emission and (3) Cy3 excitation and visualization of Cy5 emission (FRET channel), where the Cy3 and Cy5 channels are appropriate for SCy3 and Alexa647, the dyes used on our γPNA miniprobes. The Nikon NIS element advanced software was used to acquire the images. Exposure times and look up table settings were identical for all imaging conditions. For the interphase nuclei and metaphase chromosomes a series of z-stacked images (0.20-micron steps) were acquired for each and processed by deconvolution using Nikon NIS elements software (Nikon, Version 4.6, Melville, NY, USA) to remove out-of-focus light. A maximum intensity projection of each image was obtained using the Nikon NIS elements ND processing software and was analyzed for fluorescence signal intensity.

### 4.10. γPNA Fluorescence Intensity Measurements

The fluorescence intensity of γPNA stained telomere foci in interphase nuclei and metaphase chromosomes were measured by detecting objects and quantitating fluorescence intensity units in Nikon NIS Elements. A consistent minimum object threshold was established across each experiment. The total sum of intensity per object was measured.

### 4.11. Statistical Analyses

Statistical significance was calculated using Prism 6 (GraphPad Software Inc., La Jolla, CA, USA) and two-tailed unpaired Student’s *t*-test, using a 95% confidence level.

## Figures and Tables

**Table 1:** γPNA miniprobe and DNA oligomer sequences.

Name	Sequence (C-to-N for γPNA)
SCy3-γPNA	H_2_N–TCC CAA TCC–SCy3
X647-γPNA	H_2_N–CAA TCC CAA–X647
Telo-3 DNA	5′–(AGG GTT)_3_–3′
Telo-4.5 DNA	5′–(GTT AGG)_4_ GTT–3′
9-mer A DNA	5′–AGG GTT AGG–3′
9-mer B DNA	5′–GTT AGG GTT–3′

## Supplementary Materials

Supplementary materials are available online. γPNA synthesis procedure, Figures S1–S4.
