# Supplementary material for: γPNA FRET Pair Miniprobes for Quantitative Fluorescent In Situ Hybridization to Telomeric DNA in Cells and Tissue
Source: Molecules. 2017 Dec 2;22(12):2117. doi: 10.3390/molecules22122117 (PMC5895088; doi:10.3390/molecules22122117)
Supplement: Supplementary file 1 [file molecules-22-02117-s001.pdf]

Supporting Information for

γPNA FRET Pair Miniprobes for Quantitative Fluorescent in Situ Hybridization to Telomeric DNA in Cells and Tissue

Orenstein, *et al*

Page S2............ γPNA synthesis procedure

Page S3............ HPLC chromatogram for SCy3-γPNA

Page S4............ Q-TOF mass spectrum for SCy3-γPNA

Page S5............ HPLC chromatogram for X647-γPNA

Page S6............ Q-TOF mass spectrum for X647-γPNA

γPNA Synthesis

All γPNAs were synthesized on an Intavis automated peptide synthesizer using Fmoc solid phase peptide synthesis protocol on rink amide tentagel resin. This was a multistep synthesis in which each step had three major cycles, (1) removal of Fmoc group, (2) addition of the monomers and (3) capping of the unreacted sites. Between each cycle resin was washed five times with DMF. 15 mg resin (2 umol) was placed in the reaction column and swelled with DCM for 15 min. The resin was then treated twice with 400 µL of 20% piperidine/DMF for 5 min each to remove the Fmoc group. After five washes with DMF, 14 mmol of 0.2M monomer (PNA monomer or SCy3), 28 mmol of 0.3M DIEA and 13 mmol of 0.2M HATU in anhydrous DMF were added and the mixture was stirred for 30 min. The reaction mixture was then drained and washed five times with DMF and then 400 µL of capping solution (5% acetic anhydride and 6% lutidine in DMF) was added and the mixture was stirred for 5 min. These three cycles were repeated until the end of the sequence and then resin was washed five times with DCM and then dried. Crude γPNAs were obtained by treating the dried resin with TFA(95): m-Cresol(5) for 2 h, then filtered and subsequently precipitated by adding cold diethyl ether. After two washes with diethyl ether, crude γPNAs were dissolved in approx. 2 mL of 50:50 water and acetonitrile mix and prepared for lyophilization. After lyophilization, crude γPNAs were dissolved in 1 ml of 5% acetonitrile: water mixture and run on Thermo analytical HPLC system to get a crude analytical profile and then subsequently on Thermo Preparative HPLC system to purify the crude γPNAs accordingly. Finally, the fractions were pooled and analyzed via Thermo analytical HPLC as well as on Waters-Q-TOF to confirm the identity of the γPNAs and subsequently lyophilized to get pure γPNAs.

SCy3 Labeling

SCy3 labeling of γPNA was done on resin using SCy3 carboxylic acid and standard coupling conditions. The dye-labeled γPNA was cleaved from the resin and purified by HPLC.

Alexa 647 (X647) Labeling

Due to the higher cost of Alexa647, we labeled the second γPNA 9-mer miniprobe after cleavage from the resin using the following protocol:

Required Materials

- **Solution A**: PBS buffer (Phosphate-Buffered Saline, pH 7.4): Dissolve 8 g NaCl, 0.2 g KCl, 1.44 g $Na_2HPO_4 \cdot 2\ H_2O$, and 0.24 g KH_2PO_4, in 1 liter distilled water.
- **Solution B**: 0.2 M sodium bicarbonate solution, adjusted to pH 9.0 with 2 M sodium hydroxide.
- **Solution C**: To 20 parts of **Solution A** add 1 part of **Solution B** to obtain a labeling buffer of pH 8.3. Kept in an air-tight bottle, this solution will be stable for a long period of time.
- **Solution D**: Dissolve 1.0 mg of Alexa 647 NHS-ester in 50 µL of anhydrous, amine-free DMSO.

Conjugate Preparation

- Dissolve 200 nmol of unlabeled γPNA probe in 75 μL of **Solution C**. Add **Solution D** prepared above to this unlabeled probe solution.
- Incubate the reaction mixture protected from light overnight at 50 degree C.
- This reaction mixture was directly injected to prep HPLC for purification. The pure X647-γPNA was characterized by analytical HPLC and Q-TOF.

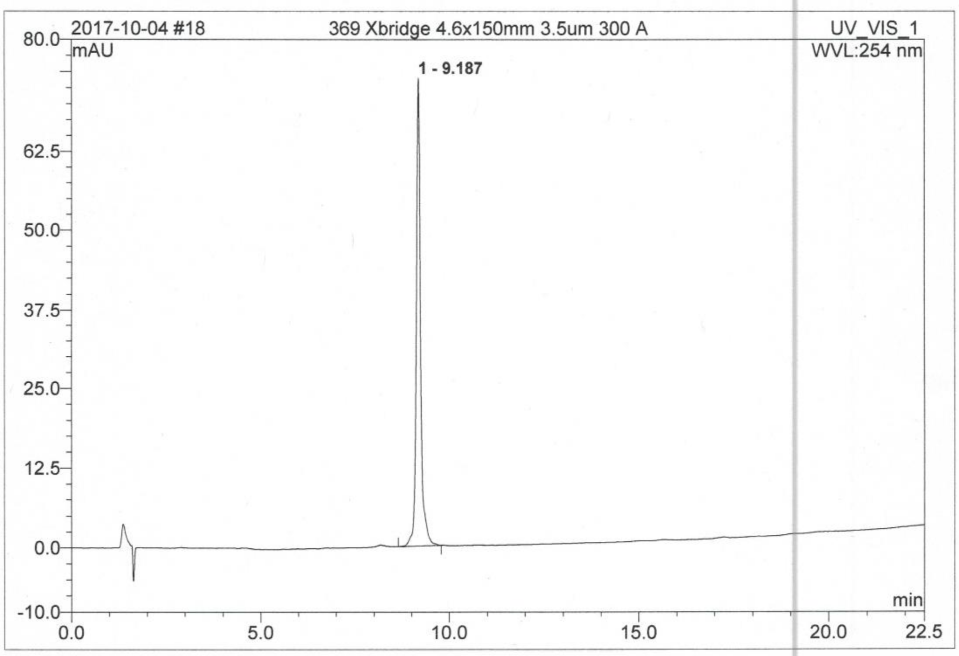

No.	Ret.Time min	Peak Name	Height mAU	Area mAU*min	Rel.Area %	Amount	Type
1	9.19	n.a.	73.776	9.777	100.00	n.a.	BMB
Total:			73.776	9.777	100.00	0.000	

Figure S1. HPLC chromatogram for SCy3-γPNA

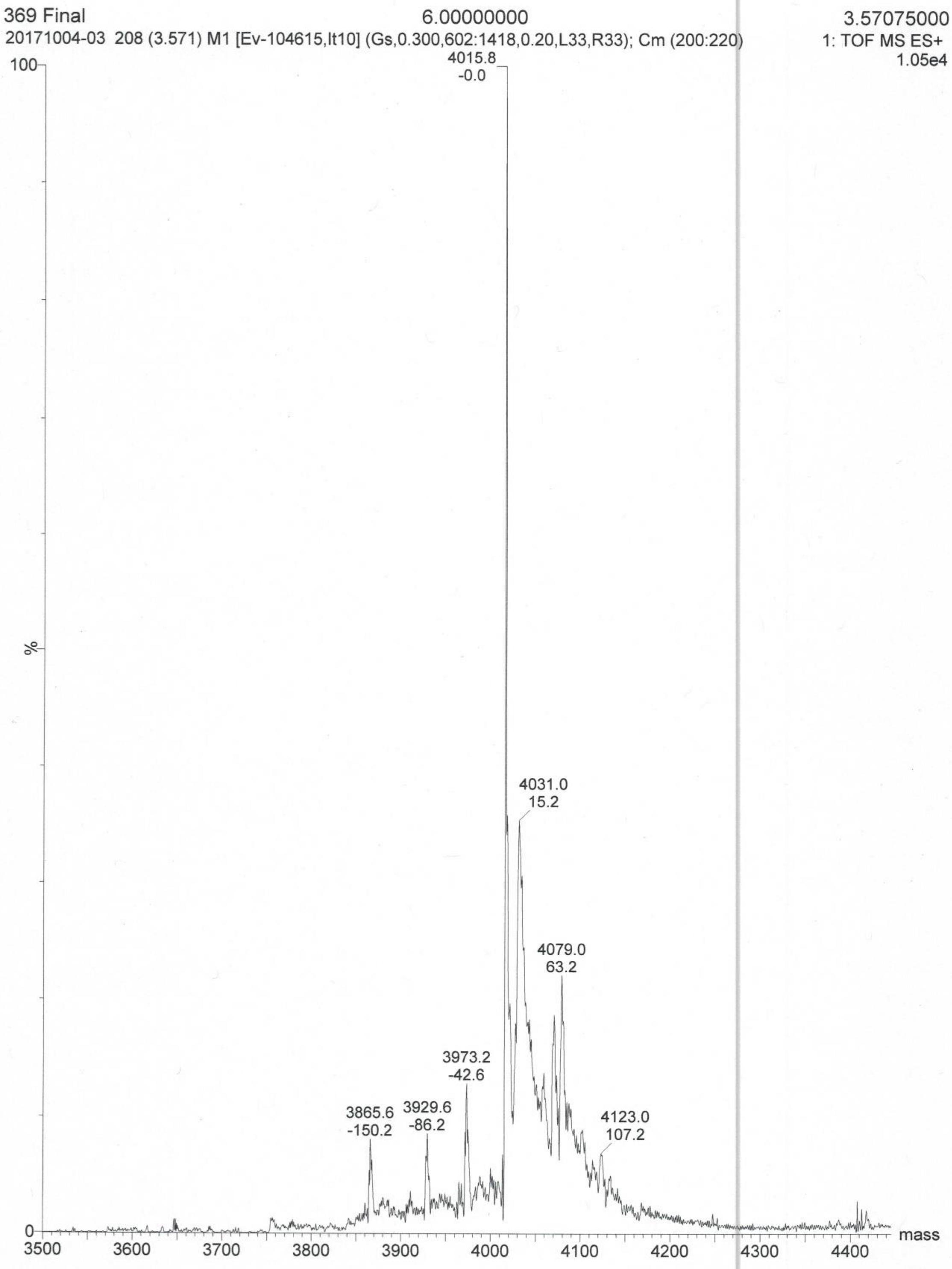

Figure S2. Q-TOF Mass Spectrum for SCy3-γPNA

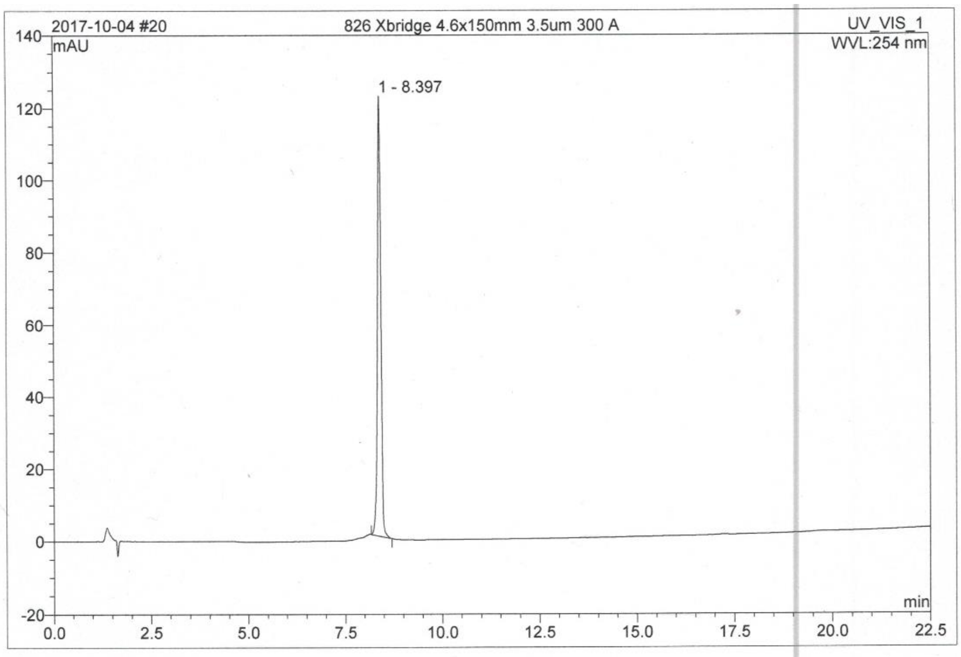

No.	Ret.Time min	Peak Name	Height mAU	Area mAU*min	Rel.Area %	Amount	Type
1	8.40	n.a.	122.128	12.564	100.00	n.a.	BMB
Total:			122.128	12.564	100.00	0.000	

Figure S3. HPLC Chromatogram for X647-γPNA

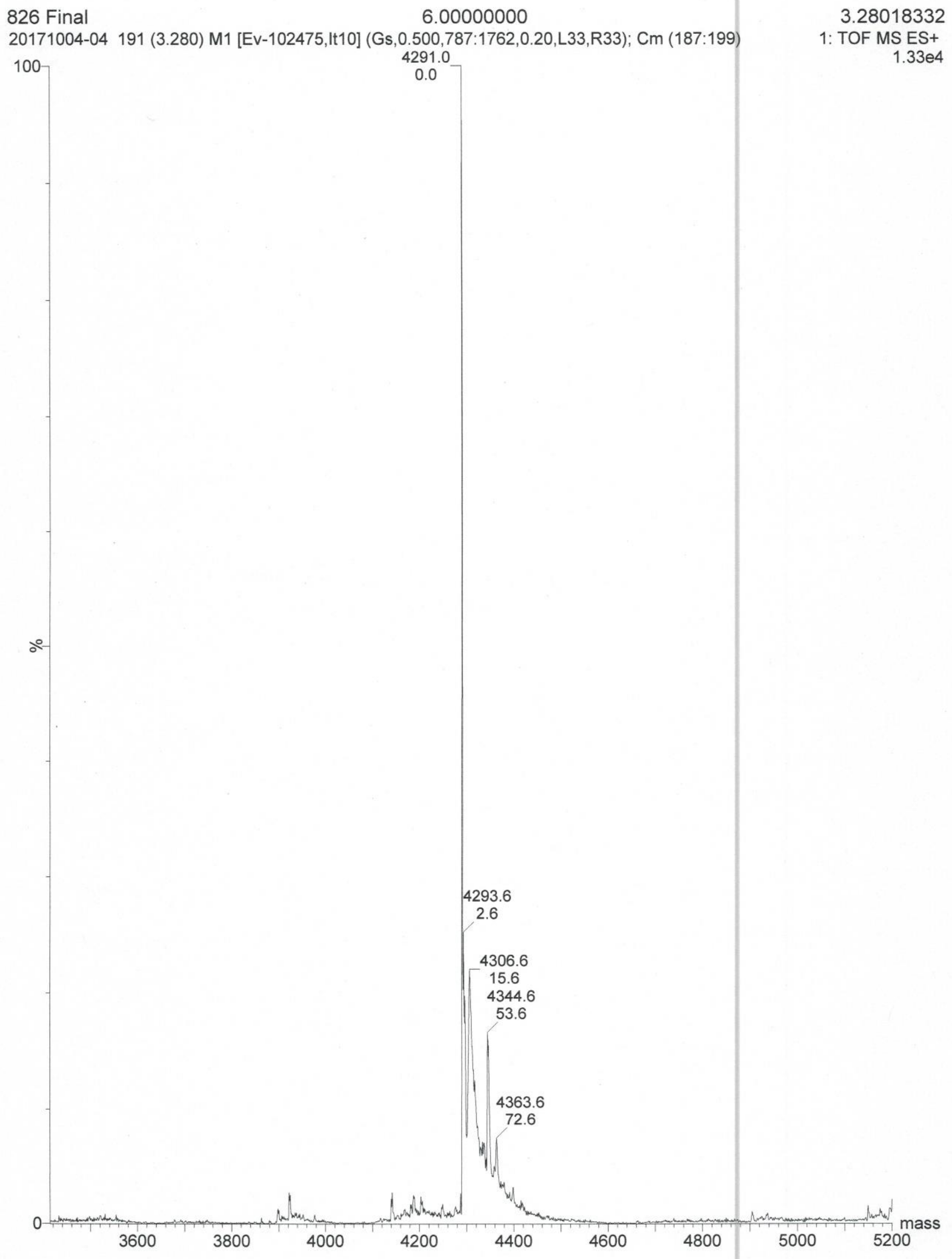

Figure S4. Q-TOF Mass Spectrum for X647-γPNA
